# Shape Variation in Neotropical *Cytheridella* (Ostracoda) Using Semilandmarks-Based Geometric Morphometrics: A Methodological Approach and Possible Biogeographical Implications

**DOI:** 10.1371/journal.pone.0168438

**Published:** 2016-12-15

**Authors:** Claudia Wrozyna, Thomas A. Neubauer, Juliane Meyer, Werner E. Piller

**Affiliations:** 1 University of Graz, Institute of Earth Sciences, NAWI Graz Geocenter, Graz, Austria; 2 Geological-Paleontological Department, Natural History Museum Vienna, Vienna, Austria; 3 Justus Liebig University, Department of Animal Ecology and Systematics, Giessen, Germany; Institute of Tibetan Plateau Research Chinese Academy of Sciences, CHINA

## Abstract

Geometric morphometrics offer effective methods to obtain information about shape and shape variability. In ostracodology, landmark-based methods are, however, not well established. To test the applicability of geometric morphometric analyses for ostracods, we investigated shape variation among recent and fossil populations of the genus *Cytheridella* using a combination of landmarks and semilandmarks. The study focuses on the species' intraspecific morphological variability on a supra-regional scale, comparing living populations from Florida, Yucatán, Colombia and Brazil. We performed Generalized least-squares Procrustes Analysis on 508 adult and juvenile specimens (valves) including stages A-1 to A-4. The analyses show that the primary pattern in shape variation is ontogenetic allometry, supporting a clear separation of adults and juveniles. Shape changes are relatively small during ontogeny from A-4 to A-1. Greatest modification of valve shape occurs during the last molt phase. Insufficient differentiation of sexes is caused by females with less developed brood pouches. Disentangling size- and non-size-dependent shape changes reveals regional differences between populations of the species *C*. *ilosvayi* and supports its taxonomic distinction from a fossil relative (*C*. *danielopoli*). The distribution of regional morphotypes of *C*. *ilosvayi* in Florida, Mexico, and Brazil are congruent with fly ways of water birds.

## Introduction

Morphometry is the quantitative description, analysis, and interpretation of shape and shape variation in biology [1–3]. Superior to the use of traditional measurements, geometric morphometric analyses purely address shape, i.e., the properties of an object that are invariant to scale, orientation and spatial position [3]. Two main approaches are available, one focusing on point-data, one on outlines and surfaces. Geometric morphometric analyses approach shape via a configuration of clearly defined homologous points in 2D or 3D space, the landmarks [3, 4]. Outline-based morphometric analyses, on the other hand, deal with open or closed curves or curve segments and largely neglect information from homologous points [5–8]; optionally, the methods can be expanded towards surfaces (e.g., [9–11]).

Numerous studies have investigated morphological variability in ostracods using morphometric analyses (e.g., [12–22]). Amongst the different outline methods available, Fourier analysis, also known as spectral analysis or harmonic analysis, is a powerful biometric tool that is particularly suited for the description of organisms lacking many homologous landmarks [5, 7, 23]. It splits a closed curve (such as the ostracod outline) into separate components or harmonics, i.e., a combination of sine and cosine waves, each one described by a number of coefficients which can be further compared among the specimens [7, 20, 24–26]. Another outline-based method, the Eigenshape analysis, has been tested for ostracodes as well [27, 28]. That method decomposes an outline into a pre-defined number of points and analyzes the angular offset between successive points [6, 9]. During the last few years B-spline analyses have become more popular [18, 29–31]. The underlying algorithm fits B-splines into the digitized outline based on a specified number of so-called “control points” [32]. Landmark-based morphometric techniques, in contrast, are not well integrated in ostracodology at present, mainly because of the lack of sufficient homologous points in most non-marine taxa [33]. As a consequence, most morphometric studies on ostracods would focus purely on outlines and neglect considerable parts of the shape variation.

To overcome the insufficiency of addressing only a part of the morphological spectrum the method of sliding semilandmarks was developed [2, 10, 11, 34]. Semilandmarks offer a convenient way to quantify two- or three-dimensional curves and surfaces, and to analyze them together with landmarks [11].

To test the applicability of geometric morphometrics using a combination of landmarks and semilandmarks for ostracods, we investigated shape variation among recent and fossil populations of the neotopical genus *Cytheridella* Daday, 1905. As for many other organisms in the Neotropics, there is a huge lack of knowledge about ostracod diversity, distribution and ecology, although they fulfill many criteria as good bio-indicators. They are abundant, are referred to respond to environmental changes in an ascertainable way, have wide geographic distributions, and sampling techniques are very cost-effective.

The genus *Cytheridella* (Limnocytheridae, Timiriaseviinae) first appeared during the early Oligocene in North America (Swain, 1999) and occurred in South America during the Miocene (*C*. *danielopoli*) [35]. Today, the genus is documented from the Americas and Africa [36, 37]. Recent American representatives comprise only the two species *C*. *ilosvayi* Daday, 1905 and *C*. *boldii* Purper, 1974. While the latter one has been reported only from its type locality, the Lake Valencia in Venezuela, *C*. *ilosvayi* is found across a huge geographic area, reaching approximately from 30°N to 30°S (see [38] for details). Earlier studies based on traditional morphometrics of soft- and hard-part morphology of *C*. *ilosvayi* from Brazil revealed the presence of distinct morphotypes [38]. In addition, *Cytheridella* species show similar valve morphologies especially at juvenile stages. This can hamper a proper identification of species at all instars.

Using geometric morphometrics, we aim to investigate the species' intraspecific morphological variability on a supra-regional scale, comparing living populations from Florida, Yucatán, Colombia and Brazil. Also, we included fossil *C*. *danielopoli* to the dataset to test for the ability of the method to differentiate between morphologically similar species. Specific questions to be addressed by this study are: can we differentiate ontogenetic stages and sexes? How does shape and size interrelate in these ostracods? Moreover, can we differentiate recent *C*. *ilosvayi* and fossil *C*. *danielopoli* at all growth stages using geometric morphometrics?

## Material

**Fig 1:**
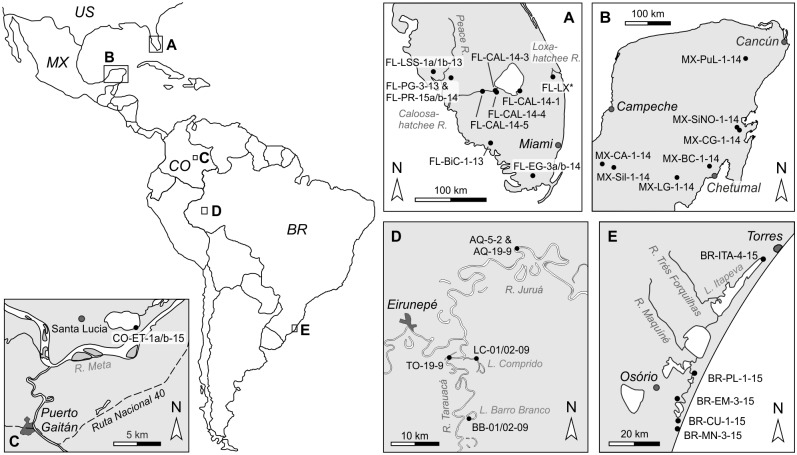
Map of sampling localities of *Cytheridella*. Code numbers as in Table 1. In map A locality FL-LX* refers to samples FL-LX-1-14 to FL-LX-6-14.

Subject of the study were the *Cytheridella* species *C*. *ilosvayi* Daday, 1905 and *C*. *danielopoli* Purper, 1979. Recent *C*. *ilosvayi* (type species of *Cytheridella*) have been sampled in several environments on public land in Florida, Mexico and Brazil (Fig 1) during field campaigns during 2009 to 2015. No *Cytheridella* species is listed in the IUCN Red List of Threatened Species (http://www.iucnredlist.org/) [39]. No permits were required for the described study, which complied with all relevant regulations.

In order to study similarities between extant *Cytheridella* and fossil relatives, we included *C*. *danielopoli* into the analyses. The fossil valves of *C*. *danielopoli* derive from late Miocene freshwater deposits at Eirunepé, SW Amazonas state, Brazil. Studied specimens were obtained by W. E. Piller and Martin Gross from the two outcrops Torre da Lua and Aquidaba (see [35, 40]. All specimens are housed in the collection of the Museu Paraense Emílio Goeldi, Belém (Inv. No. MPEG-90-M to MPEG-193-M), additional material is stored at the Universalmuseum Joanneum, Department for Geology & Palaeontology, Graz (Inv. No. UMJG&P 210.903). All necessary permits were obtained for the described study, which complied with all relevant regulations. Permits for the fieldwork were issued by Conselho Nacional de Desenvolvimento Científico e Tecnológico/Ministério da Ciência e Tecnologia (CNPq/MCT; process number EXC 010389/2009-1).

Information on sampling localities is summarized in Table 1.

**Table 1:** Sampling localities with information about sample codes, locality names, country, sampling date and coordinates. The fossil *C*. *danielopoli* derived from fossil sites are referred to outcrops Aquidaba (AQ) and Torre da Lua (TO). For details see [35, 40].

Sample	Locality	Date	Country	N (S)	E (W)
BB 01/02 09	Barro Branco	27.09.2009	Brazil	06°50’18.3”	69°45’37.0”
LC 01/02 09	Lago Comprido	27.09.2009	Brazil	06°43’52.7”	69°44’33.9”
BR-CU-1-15	Custódia Lagoon	04.09.2015	Brazil	30°02'15.2''	50°10'20.2''
BR-MN-3-15	Rio de Relógio	04.09.2015	Brazil	30°04'10.3''	50°12'20.8''
BR-PL-1-15	Passos da Lagoa	03.09.2015	Brazil	29°51'16.1''	50°06'57.9''
BR-ITA-4-15	Itapeva Lagoon	06.09.2015	Brazil	29°22'32.6''	49°47'39.2''
BR-EM-3-15	Emboaba Lagoon	03.09.2015	Brazil	29°57'52.8''	50°13'27.4''
CO-ET-1a-15	Estero Texas	04.02.2015	Colombia	04°24'31.2''	71°58'44.5''
CO-ET-1b-15	Estero Texas	04.02.2015	Colombia	04°24'31.2''	71°58'44.5''
FL-PG-3-13	Shell Creek, Peace River	28.11.2013	Florida, US	26°58'26.99''	81°53'21.8''
FL-LSS-1a/1b-13	Little Salt Spring	26.11.2013	Florida, US	27°4'29.3''	82°14'.37''
FL-BiC-1-13	Big Cypris National Reserve	29.11.2013	Florida, US	25°53'29.53''	81°16'14.52''
FL-LX-1-14	Loxahatchee River	31.07.2014	Florida, US	26° 56'03.0''	80°10'36.4''
FL-LX-2-14	Loxahatchee River	31.07.2014	Florida, US	26° 56'32.5''	80°10'19.2''
FL-LX-3-14	Loxahatchee River	31.07.2014	Florida, US	26°56'40.28"	80°10'15.94"
FL-LX-4-15	Loxahatchee River	31.07.2014	Florida, US	26°56'46.91''	80°10'15.42''
FL-LX-5-15	Loxahatchee River	31.07.2014	Florida, US	26°26'49.8''	80°10'12.4''
FL-LX-6-15	Loxahatchee River	31.07.2014	Florida, US	26°56'52.5''	80°10'11.1''
FL-CAL-14-1	Caloosahatchee River	06.08.2014	Florida, US	26°43'37.9''	80°42'10.5''
FL-CAL-14-3	Caloosahatchee River	06.08.2014	Florida, US	26°50'22.4''	81°04'51.8''
FL-CAL-14-4	Caloosahatchee River	06.08.2014	Florida, US	26°50'09.8''	81°05'14.4''
FL-CAL-14-5	Caloosahatchee River	06.08.2014	Florida, US	26°47'21.7''	81°18'33.6''
FL-PR-15a/b-14	Shell Creek, Peace River	08.08.2014	Florida, US	26°58'26.99"	81°53'21.81"
FL-EG-3a/b-14	Everglades	02.08.2014	Florida, US	25°26'2.0''	80°45'12.3''
MX-CA-1-14	Cenote Azúl	12.08.2014	Mexico	18°48'43.3''	90°38'48.1''
MX-SiNo-1a-14	Siiji No-Ha Cenote	11.08.2014	Mexico	19°28'33.5''	88°03'15.6''
MX-CG-1-14	Cenote Galeana	11.08.2014	Mexico	19°27'45.6''	88°1'46.3''
MX-BC-1a-14	Laguna Bacalar	11.08.2014	Mexico	18°39'5.8''	88°24'33.07''
MX-Pul-1-14	Punta Laguna	13.08.2014	Mexico	20°38'49.4''	87°38'04.1''
MX-LG-1-14	Lake "Las Garantias"	13.08.2014	Mexico	18°27'11.7''	89°00'42.0''
MX-Sil-1-14	Silvituc	12.08.2014	Mexico	18°38'29.8''	90°16'28.1''
AQ 5 2	Aquidaba		Brazil	06°31'40.8''	69°39'52.0''
AQ 19 9	Aquidaba		Brazil	06°31'40.8''	69°39'52.0''
TO 19 9	Torre da Lua		Brazil	06°49'23''	69°47'04''

In order to exploit as much morphological information as possible, we used left and right valves from adults and instars, including stages A-1 to A-4 (Table 2). In total, 508 valves were used for the analyses (247 right valves [RV], 261 left valves [LV]). Samples from Brazil (BB 01 09, LC 01 09, BR-ITA-4-15, CO-ET-1a/b-2015) contained exclusively carapaces with soft parts that enabled to use left and right valves from the same specimens.

**Table 2:** Overview about the analyzed valve material. Indicated is the number of moult stages of each sample. Asterisks refer to specimens of A-4.

sample	females		males		A-1		A-2		A-3/4		
	LV	RV	LV	RV	LV	RV	LV	RV	LV	RV	
BB 01/02 09	7	6	1				1	1			16
LC 01/02 09	10	9	2	2	4	5			1	1	34
BR-CU-1-15	2										2
BR-MN-3-15			1								1
BR-PL-1-15				1							1
BR-ITA-4-15	14	14	6	7							41
BR-EM-3-15	1	1									2
CO-ET-1a-15	1	2	1								4
CO-ET-1b-15	2		1	1							4
FL-PG-3-13	2	3	1		1						7
FL-LSS-1a/1b-13		1	1								2
FL-BiC-1-13		1									1
FL-LX-1-14	4	4			1	1					10
FL-LX-2-14					4	3		1			8
FL-LX-3-14	1	3	2	1	2	2		1			12
FL-LX-4-15	3	1			1	2					7
FL-LX-5-15			1	1							2
FL-LX-6-15		1			1	1					3
FL-CAL-14-1	2	2	1	1							6
FL-CAL-14-3	5	5	3	2	3	2	3	2	3	4	32
FL-CAL-14-4	4	3	1	4	2	1					15
FL-CAL-14-5	2	1	3	1							7
FL-PR-15a/b-14	1	3	1		1	3			1	1	11
FL-EG-3a/b-14	4	2	1	3	5	3	2	3			23
MX-CA-1-14	10	8	4	4	4	4	1				35
MX-SiNo-1a-14	3	4	3	4	5	1		1		1	22
MX-CT-1-14	1	1									2
MX-CG-1-14	0	1	2	1	1	1					6
MX-BC-1a-14	8	5	3	6	1		4	2		1	30
MX-Pul-1-14	10	14	3	4	6	4				2	43
MX-LG-1-14	3	7	3	2	1		1				17
MX-Sil-1-14	1	1	1	1							4
AQ 19 9	6	6	2	2	4	7	1	4	4	2 (1)*	39
AQ 5 2	3	2	2	1	6	1	1	5	3	3 (2)*	29
TO 19 9	0	0	0	1	2	5	12	4	4 (1)*	1	30
	110	111	50	50	55	46	26	24	17	19	508

## Methods

### Generalized Procrustes Analysis and Multivariate Statistics

**Fig 2:**
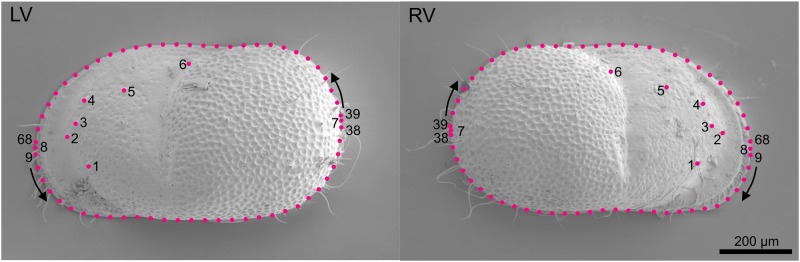
Landmark configuration. Landmark configuration on left and right valves of *Cytheridella*.

Eight points were chosen as landmarks (LM; Fig 2). LMs 1–5 are type-I landmarks and characterize anterior pore tubuli; LM 6 is a type-II landmark defined by the dorsal dip point of the posterior curvature; LMs 7–8 are type-III landmarks delimitating the maximum anterior and posterior curvature. The valve outline was defined by two curves between LMs 7–8, each comprising 30 equidistantly spaced semilandmarks (Fig 2). Both landmarks and semilandmarks were set on digitized images with the program TpsDig v. 2.17 [41]. Right and left valves were investigated separately due to dimorphism in size and shape (adont hinge, left valves more bulgy; [36]). The sliders file to determine the sliding direction of the semilandmarks was created in TpsUtil v. 1.58 [42]. A generalized least-squares Procrustes Analysis was performed in TpsRelw v. 1.53 [43]. The analysis computes the consensus configuration, the partial warps, the relative warps, and centroid size. The semilandmarks are allowed to slide along the curves in order to minimize the bending energy between each specimen and the Procrustes average shape [11, 44]. The deviations of the (semi)landmark configurations from the consensus were visualized by thin-plate spline deformation grids.

Principal Components Analysis (PCA), i.e. the Relative Warps Analysis, was used to explore the morphospaces and Multivariate Analysis of Variance (MANOVA) and Canonical Variates Analysis (CVA) to test for significant differences between pre-defined groups (ontogeny/sex/species). CVA fundamentally differs from PCA in that it requires specimens to be assigned to pre-defined groups, and then tests how well the data (e.g., warp scores) can be used to support those assignments. It aims at maximizing the ratio of the between-group variance to the within-group variance. Axes are scaled according to patterns of within-group variation and are not simple rotations of the original coordinate system as in PCA. Consequently, the distance between specimens or groups in a CVA-morphospace is not necessarily equal to their distance in the original morphospace (for details on the method see [4, 45]). Since CVA is easily biased if the number of variables exceeds the number of specimens per group [45], we used for the computation the 15 first relative warps, which account for c. 97% of the shape variation in both valves. Due to low individual numbers *C*. *danielopoli* males were excluded. P-values of pairwise comparisons in MANOVA were Bonferroni-corrected.

In addition, we tested for morphological differences between populations from different geographic regions (and stratigraphic ages). To achieve a reasonable and statistically reliable comparison, we chose only female adult specimens for each valve, offering the highest number of specimens across all ontogenetic stages. The CVA was based on the 20 first relative warps resulting from the Generalized least-squares Procrustes Analysis.

MANOVA and CVA were computed in PAST v. 2.17c [46].

### Shape-Size Relationships

The geometric morphometric analysis disregards individual size. However, because of their allometric growth [47], morphological variability among the ostracods is to some extent expected to reflect size differences, especially with respect to differences between ontogenetic stages. To test for such a potential shape-size relationship, we performed ordinary least-squares regression analyses of valve (log 10 transformed) centroid sizes onto the first three relative warps for each valve in PAST.

## Results

### Relative Warps Analysis

**Fig 3:**
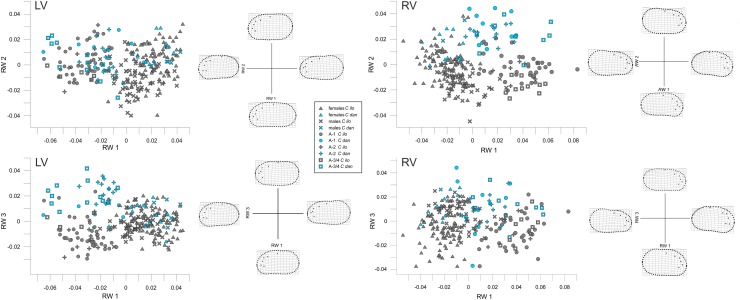
Relative warps analysis. Relative warps analysis of left and right valves (LV and RV, respectively) of the first three warps and the associated thin-plate splines at maximum and minimum scores.

The relative warps analysis reveals, despite minor overlap, a clear distinction between adult and juvenile specimens in both left and right valves. Shape variations explained by the first three relative warps (RW) are 43.9%, 12.4% and 10.7% for left valves and 41.4%, 15% and 12.8% for right valves. Thin-plate spline grids for the minimum and maximum values and the outlier between axes of each of the first three RWs are displayed in Fig 3.

Shape variation along RW 1 is primarily related to outline shape and the relative positions of the pore conuli (LMs 1–5). Positive scores correspond to enlarged posterodorsal shapes and a narrow arrangement of pore conuli. Along RW 2, positive values reflect more posterodorsally rounded and slightly convex ventral outlines. Negative values are associated with a decrease in the curvature of the posterodorsal shape as well as slight concave bend of the ventral outline. In left valves, RW 2 primarily differentiates between elongated (negative scores) and shortened valves (positive scores). LMs 1 to 6 play a minor role. In right valves, RW 2 parallels variation of the general shape (elongated with anterior inflation versus shortened with posterior inflation) as well as the relative positions of LMs 2–4 (pore conuli 2 to 4). In particular, LM 2 and 3 are closer in elongated valves, and more distant from each other in shortened valves.

Shape variation associated with RW 3 is also slightly different for left and right valves. Variation of left valves ranges from a convex (positive scores) to concave (negative scores) ventral shape. Moreover, the distance between pore conuli 2–4 varies. Especially LM 2 varies between a downwards shifted position and, thus, increasing distance to LM 3 (negative scores) and a closer position to LM 3 (positive scores). For right valves, in contrast, RW 3 reflects outline variation between shortened (positive scores) and elongated (negative scores) shapes. In addition, the position of the dorsal dip point of the posterior curvature (LM 6) varies between proximal (positive) and marginal (negative).

The distributions of the specimens show clear patterns only along RW 1 with respect to ontogeny. Adult individuals are largely separated from juveniles occupying higher positive (left valves) and negative scores (right valves), respectively. Juveniles are not clearly sorted according to their moult stage. Females and males overlap. Along RW 2, shape variation of females reflects different outline shapes ranging from forms with a well-developed brood pouch (positive scores, left and right valves) to elongated and less-developed brood pouches (negative scores, left and right valves). Males are characterized by negative scores reflecting their less curved posterodorsal shapes.

Morphological differences between *C*. *ilosvayi* and *C*. *danielopoli* are not discernible along RW 1, and their expression differs between left and right valves. The best differentiation between both species is achieved along RW 3 in left valves and along RW 2 in right valves. In both cases, the degree of separation increases with decreasing ontogenetic age: the two species are almost perfectly separated during stages A-3/4 and A-2, while they overlap in stages A-2/A-1 and adult specimens. Only in right valves, males of both taxa seem to differ, but the low number of male *C*. *danielopoli* (n = 2) does not allow a reliable conclusion.

In summary, shape variation along RW 1 mainly corresponds to ontogenetic differences, while the differences between the two species are represented by RW 2 (right valves) and RW 3 (left valves).

### Canonical Variates Analysis

**Fig 4:**
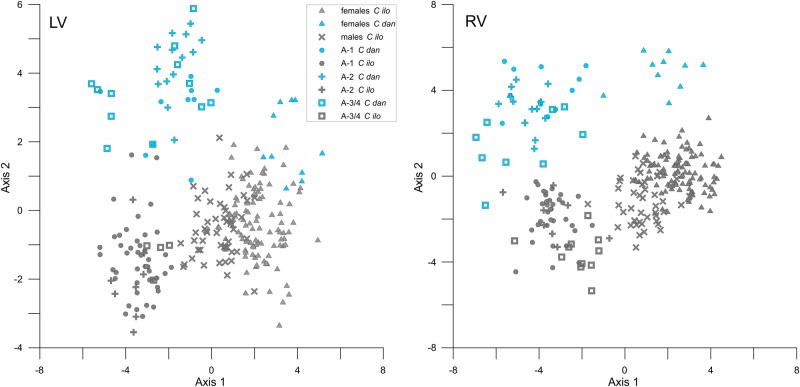
Canonical variate analyses of adults and juveniles. Analyses are based on the relative warps scores of females, males, and juveniles of *C*. *danielopoli* and *C*. *ilosvayi* including A-1, A-2, and A-3/4. Due to small specimens number, males of *C*. *danielopoli* were excluded from this analysis.

The CVA based on the first fifteen relative warps of the whole dataset achieved partial delimitations of ontogenetic stages for both valves. The overall MANOVA statistics confirmed that the group means are significantly different (LV: Wilks’ lambda test: Λ_Wilks_ = 0.0081, F = 19.19, p < 0.001, Pillai’s trace test: Λ_Pillai_ = 2.81, F = 11.94, p < 0.001; RV: Wilks’ lambda test: Λ_Wilks_ = 0.0051, F = 21.01, p < 0.001, Pillai’s trace test: Λ_Pillai_ = 2.86, F = 11.77, p < 0.001). Adult males and females each form well-defined clusters with little overlap (LV: p < 0.001; RV: p < 0.001) and are also distinct from juvenile stages. Species are clearly separated with significantly different group means (Fig 4). Among the juveniles, however, delimitation of stages differs between both valves and species.

While the earliest stage of *C*. *danielopoli* (A-3 plus A-4) forms a more or less distinct cluster in right valves, they cannot be significantly distinguished from stage A-2 and A-1 in left valves (p = 1, p = 0.20, respectively). Earliest juveniles of *C*. *ilosvayi* can be differentiated significantly from A-1 in both valves (LV: p = 0.002; RV: p < 0.001), but not from A-2 (LV: p = 0.53; RV: p = 1).

Stages A-1 and A-2 overlap in both valves of both species, but only in left valves of *C*. *danielopoli* the group means are not significantly different (p = 1). All other pairwise comparisons between group means yielded significant differences.

### Geographic and Interspecific Shape Variation in Female Specimens

In addition to the analyses based on the total dataset, we tested for differences of the morphological variability among populations of different geographic regions as well as between the living *C*. *ilosvayi* and the fossil *C*. *danielopoli*.

**Fig 5:**
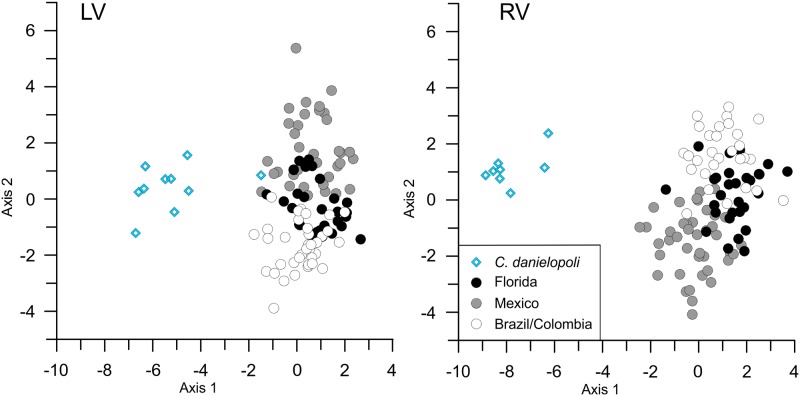
Canonical variate analyses of female *C*. *danielopoli* and *C*. *ilosvayi*. Analyses are based on the relative warps scores of females including *C*. *danielopoli* and *C*. *ilosvayi* from Mexico, Florida, Brazil and Colombia.

Except for a single outlier in left valves, there is no overlap between the species (Fig 5). The outlier represents a specimen with a short posterior part. The analysis indicates a broader morphological variability for *C*. *ilosvayi* than for *C*. *danielopoli*. Populations of *C*. *ilosvayi* from Brazil, Florida and Mexico are discriminated along the second canonical axis. While Brazilian and Colombian females overlap with populations of both Florida and Mexico, the latter two regions are almost completely separated.

The MANOVA confirmed that the group means are significantly different, regarding the overall statistics (LV: Wilks’ lambda test: Λ_Wilks_ = 0.058, F = 9.261, p < 0.001; Pillai’s trace test: Λ_Pillai_ = 1.739, F = 8.19, p < 0.001; RV: Λ_Wilks_ = 0.028, F = 12.77, p < 0.001, Λ_Pillai_ = 1.974, F = 10.83, p < 0.001) as well as the pairwise comparisons.

### Shape-size Relationships

**Fig 6:**
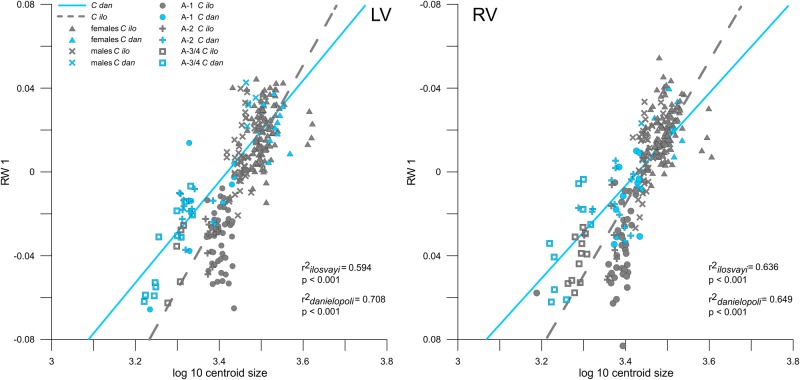
Regression analyses. Regressions of valve size (given as centroid size) and the first relative warp for left (LV) and right valves (RV) based on the entire dataset.

The linear regressions of RW 1 with centroid size yielded comparably strong relationships for both valves (Left valves: r^2^_ilosvayi_ = 0.594, p < 0.001, r^2^_danielopoli_ = 0.708, p < 0.001; Right valves: r^2^_ilosvayi_ = 0.636, p < 0.001, r^2^_danielopoli_ = 0.649, p < 0.001; Fig 6). Hence, shape variation along RW 1 is between 60–70% explained by size differences. This result is hardly surprising given the order of ontogenetic stages along this warp as seen in the Relative Warps Analysis already. The strength of the shape-size relationship is for both valves lower in *C*. *ilosvayi*. There is distinctly less shape-size relationship along RW 2 (LV: r^2^_ilosvayi_ = 0.119, p < 0.001, r^2^_danielopoli_ = 0.033, p < 0.001; RV: r^2^_ilosvayi_ = 0.112 < 0.001, r^2^_danielopoli_ = 0.054, p < 0.001) and RW 3 (LV: r^2^_ilosvayi_ = 0.184, p < 0.001, r^2^_danielopoli_ = 0.361, p < 0.001; RV: r^2^_ilovayi_ = 0.012, p < 0.001, r^2^_danielopoli_ < 0.001, p < 0.001).

The general allometric trend observed corresponds to an elongation and increase of the posterior part in relation to the anterior part. In other words, a transition occurs from juveniles with a relatively pointed posterior to the reverse shape of adults with a relatively pointed anterior. Additionally, the position of the sulcus is displaced from a posteromedian position in juveniles to a more anteromedian position in adult specimens. Thereby, the distance between the sulcus and the pore conuli is reduced. Also, the anterior pore conuli shift to a more narrow position in adult individuals.

## Discussion

### Ontogenetic Patterns

The succession of instars and sexes along the first relative warp indicates that the primary pattern in shape variation of *Cytheridella* species is the ontogenetic allometry. In general, shape change during the ontogeny of *Cytheridella* can be described as following: the anterior part of the valves is rounded in the earliest ontogenetic stage observed in this dataset, whereas the posterior part starts off more pointed (cf. Fig 3). Through ontogeny, the posterior part, more precisely the posterodorsal quadrant, enlarges. This development is traceable by LM 6, which represents the position of the transversal sulcus. Accordingly, there is a clear separation into adults and juveniles. Although the regression analyses showed that RW 1 is clearly tied to size, juveniles are not aligned according to instars along RW 1. This is also shown by the CVA (Fig 4), which illustrates similar patterns as the relative warps: adults and juveniles are separated; A-1 and A-2 overlap and A-3/4 build a distinct cluster only in right valves. In left valves all juvenile instars overlap.

This result indicates little shape change during the transition from A-4 to A-1. A comparably large morphological alteration happens during the last molt phase from A-1 to adults (Fig 4). Already previous studies have shown that the degree of shape change varies during the ontogeny of some species. The marine species *Tyrrhenocythere amnicola* (Sars, 1887) evinces greatest morphological changes between A-6 and A-5 [28]. *Bradleya normani* (Brady, 1866), in turn, shows greatest change between instars A-4 to A-3 and A-3 to A-2 [48]. Our dataset comprises juveniles only down to A-4; it is, thus, possible that distinct shape changes, which might have occurred earlier during ontogeny, remained unnoticed. In comparison, ontogenetic shape changes are similar for the two species *C*. *danielopoli* and *C*. *ilosvayi*, confirming their close relationship.

Another feature of the juveniles is their relatively high variability compared to adult specimens, and it is observable along each of the three first relative warps (Fig 3). Under environmentally controlled conditions and genetic homogeneity the cypridoidean species *E*. *virens* exhibited a similarly high individual shape variation of juveniles which decreased from A-4 onwards. It has been suggested that environmental factors play a major role in shape determination during ontogeny [49].

The insufficient differentiation of sexes in *C*. *ilosvayi* is caused by females with a less developed brood pouch, resembling the morphology of males. This result undoubtedly has important consequences in sex identification in fossil samples and indicates the need to assess traits involved in sexual dimorphism before making inferences [33]. Morphological differentiation between sexes is achieved through males which have concave ventral shapes while females display a slight depression dorsally and both, convex and concave ventral outlines. Contrasting to *C*. *ilosvayi*, sexes are not differentiated properly in *C*. *danielopol* probably due to relatively large and elongated posterior shapes of males and resembling, thus, females.

### Species Differentiation

The CVA in Fig 4 and associated MANOVA demonstrate clearly the offset between the two species compared to their intraspecific variability. Except for single outliers in left valves the two species do not overlap. In summary, *C*. *danielopoli* is characterized by more elongated shapes and straight ventral shapes. Specific differences between juveniles of the two species are observed from the relative warps analysis, where they are almost completely separated. Juveniles of *C*. *danielopoli* are also characterized by straight ventral outlines with an obtuse posterior compared to the more pointed posterior of *C*. *ilosvayi*. More juvenile specimens are needed to test at which instars species differentiation is significantly achieved.

### Geographic Shape Variability

Apart from the clear specific differences, the CVA of females also indicates geographic shape variability in *C*. *ilosvayi* (Fig 5). Interestingly, the morphological differences are higher between populations from Mexico and Florida than between those from Brazil and Florida. Although the groups have significantly different group means, the CVA indicates widely overlapping clusters. Hence, the analyses indicate the presence of distinct regional morphotypes, but a clear differentiation is not achieved. Even at the population level (female) morphotypes were found. These morphotypes are primarily discriminated by valves size but reveal also distinct differences in their soft part morphologies (i.e., podomere ratios of antennula and antenna [38]).

Further studies including males and juveniles are required to explore regional differences and allow a taxonomic classification of these morphotypes. This may ultimately help to identify biogeographical and/or phylogeographic patterns in *Cytheridella*.

For now, we can only speculate about the causes for the present disjunct distribution of *C*. *ilosvayi* because information about its origin, dispersal and fossil record are limited. Given the patchy nature of freshwater environments, long-distance dispersal in freshwater ostracods relies on passive dispersal. Several studies have stressed the importance of waterfowl-mediated dispersal of Ostracoda (e.g., [50–53]). Individuals are transported occasionally across long distances, internally or externally, often involving desiccation-resistant eggs but also living ostracods [51]. The ability to produce resting eggs is well documented from Cypridoidea but has been reported for some Limnocytheroidea, the family to which *Cytheridella* belongs, as well [54].

Accepting the preliminary hypothesis that *C*. *ilosvayi* has been dispersed via water birds, the patterns of similarities and differences of the regional morphotypes can be viewed in a different light. Instead of paralleling geographic distance, they might mirror the structure of present waterfowl flyways across the Americas (e.g., [55]). Four major routes are indicated for migrating water birds from north to south, i.e., the Pacific, Central, Mississippi and Atlantic flyways [56]. Although we lack information about the geographic origin of the species and the age of the populations, the present patterns suggest that dispersal of *C*. *ilosvayi* between Florida, Mexico and Brazil may have happened via the Mississippi or Atlantic flyways. Moreover, the disjunct distribution is likely a result of multiple independent dispersal phases. The fact that the morphological differences are higher between populations from Mexico and Florida than between those from Brazil and Florida argues for a longer geographic separation.

## Conclusion

The outcome of the present investigation shows the potential of combining characteristics of the outline and surface to study ostracod valve morphology. So far, studies have focused either solely on the ostracod outline (e.g., [14, 18, 22, 31, 57]) or on specific features of the valve (e.g., [12, 14, 16, 18, 22, 31, 48, 57]). The geometric morphometric approach applied here enabled the detailed documentation of the patterns of allometric growth in two species of *Cytheridella*, the discrimination of closely related species, as well as the differentiation of regional morphotypes in one of them. More precisely, our results indicate that ontogenetic shape variation in *Cytheridella* is largely owed to outline variation as well as the position of the transversal sulcus and the relative distance between the pore conuli. The patterns of allometric growth are similar in recent *C*. *ilosvayi* and fossil *C*. *danielopoli*: comparably small morphological change between juvenile instars is followed by a major morphological alteration during the last molt phase. While this analogy confirms the close relationship of both species, the analysis supports their discrimination based on elongated outline and straight ventral shape. Furthermore, regional shape variation is detected for *C*. *ilosvayi*, evidencing the presence of distinct morphotypes in populations from Florida, Mexico and Brazil. The populations are, however, not fully discriminated, and a taxonomic conclusion still requires the evaluation of additional data, including information from other instars as well.

The results clearly encourage the utilization of both valves in morphometric analysis, due to mutative development of shape variables in left and right valves. We hope that this study stimulates the use of geometric morphometric analyses in ostracodology. Unlike many other morphometric approaches, it supports the combination of outline and landmark features and, above all, it is an easy and quick method to address shape and shape variability.

Finally, the identification of regional morphotypes of *C*. *ilovayi* reveals a biogeographical pattern that can be interpreted to reflect water bird’s flyways across the Americas.

### Data Reporting

Data are available at the permanent web site of the Institute of Earth Sciences, University of Graz. Data include the.tps files for left and right valves and the respective results of Relative Warps Analysis and CVA. Files are readily accessible under the following URL: http://iewarchiv.uni-graz.at/zusatz/NeotropicalCytheridella/.
